# Construction of a computable cell proliferation network focused on non-diseased lung cells

**DOI:** 10.1186/1752-0509-5-105

**Published:** 2011-07-02

**Authors:** Jurjen W Westra, Walter K Schlage, Brian P Frushour, Stephan Gebel, Natalie L Catlett, Wanjiang Han, Sean F Eddy, Arnd Hengstermann, Andrea L Matthews, Carole Mathis, Rosemarie B Lichtner, Carine Poussin, Marja Talikka, Emilija Veljkovic, Aaron A Van Hooser, Benjamin Wong, Michael J Maria, Manuel C Peitsch, Renee Deehan, Julia Hoeng

**Affiliations:** 1 Selventa, One Alewife Center, Cambridge, MA 02140, USA; 2 Philip Morris International R&D, Philip Morris Research Laboratories GmbH, Fuggerstr.3, 51149 Koeln, Germany; 3 Philip Morris International R&D, Philip Morris Products S.A., Quai Jeanrenaud 5, 2000 Neuchâtel, Switzerland

## Abstract

**Background:**

Critical to advancing the systems-level evaluation of complex biological processes is the development of comprehensive networks and computational methods to apply to the analysis of systems biology data (transcriptomics, proteomics/phosphoproteomics, metabolomics, etc.). Ideally, these networks will be specifically designed to capture the normal, non-diseased biology of the tissue or cell types under investigation, and can be used with experimentally generated systems biology data to assess the biological impact of perturbations like xenobiotics and other cellular stresses. Lung cell proliferation is a key biological process to capture in such a network model, given the pivotal role that proliferation plays in lung diseases including cancer, chronic obstructive pulmonary disease (COPD), and fibrosis. Unfortunately, no such network has been available prior to this work.

**Results:**

To further a systems-level assessment of the biological impact of perturbations on non-diseased mammalian lung cells, we constructed a lung-focused network for cell proliferation. The network encompasses diverse biological areas that lead to the regulation of normal lung cell proliferation (Cell Cycle, Growth Factors, Cell Interaction, Intra- and Extracellular Signaling, and Epigenetics), and contains a total of 848 nodes (biological entities) and 1597 edges (relationships between biological entities). The network was verified using four published gene expression profiling data sets associated with measured cell proliferation endpoints in lung and lung-related cell types. Predicted changes in the activity of core machinery involved in cell cycle regulation (RB1, CDKN1A, and MYC/MYCN) are statistically supported across multiple data sets, underscoring the general applicability of this approach for a network-wide biological impact assessment using systems biology data.

**Conclusions:**

To the best of our knowledge, this lung-focused Cell Proliferation Network provides the most comprehensive connectivity map in existence of the molecular mechanisms regulating cell proliferation in the lung. The network is based on fully referenced causal relationships obtained from extensive evaluation of the literature. The computable structure of the network enables its application to the qualitative and quantitative evaluation of cell proliferation using systems biology data sets. The network is available for public use.

## Background

The immediate goal of this work was to construct a computable network model for cell proliferation in non-diseased lung. Lung epithelial cells are stimulated to proliferate upon injury as a mechanism for renewal [1]. Alterations in the control of cell proliferation play a pivotal role in lung diseases including cancer, COPD, and pulmonary fibrosis. Cancer results from both gains of inappropriate growth signaling as well as the loss of mechanisms inhibiting proliferation [2]. Hyperplasia of mucus-producing goblet cells and airway smooth muscle contribute to COPD pathology [3]. Pulmonary fibrosis is characterized by excessive proliferation of lung fibroblasts, resulting in impaired lung function [4]. Thus, increasing the molecular understanding of the regulation of cell proliferation in the lung will serve to aid in the treatment and prevention of several lung diseases.

Comprehensive and detailed pathway or network models of the processes that contribute to lung disease pathology are needed to effectively interpret modern "omics" data and to qualitatively and quantitatively compare signaling across diverse data sets. The ultimate goal of this work is to evaluate the biological impact of xenobiotics and environmental toxins on experimental systems such as lung cell cultures or whole rodent lung. Network models representing key biological processes as they occur in non-diseased cells are crucial for this effort. Tumor cell lines and other cell contexts representing advanced disease states have genetic changes and altered signaling networks that may not be present in normal, non-diseased cells. Thus, the network model described in this report is focused on biological signaling pathways expected to be functional and to regulate cell proliferation in non-diseased lung.

Many different approaches can be taken to develop biological models. Biological pathways such as those captured by KEGG (Kyoto Encyclopedia of Genes and Genomes) [5] are manually drawn pathway maps linking genes to pathways; KEGG pathways have limited computational value for analysis of systems biology data sets beyond directly mapping observed changes to pathways and assessing over-representation. Dynamic biochemical models, such as those commonly encoded in SBML (systems biology markup language) [6], are useful for assessing the dynamic behavior of biochemical systems. However, because dynamic biochemical models require a large number of parameters, they are generally limited to representation of simplified and well-constrained biological processes, and are thus not well suited to the comprehensive evaluation of complex systems consisting of multiple inter-related signaling processes.

Reverse Causal Reasoning (RCR) is a systems biology methodology that evaluates the statistical merit that a biological entity is active in a given system, based on automated reasoning to extrapolate back from observed biological data to plausible explanations for its cause. RCR requires an extensive Knowledgebase of biological cause and effect relationships as a substrate. RCR has been successfully applied to identify and evaluate molecular mechanisms involved in diverse biological processes, including hypoxia-induced hemangiosarcoma, Sirtuin 1-induced keratinocyte differentiation, and tumor sensitivity to AKT inhibition [7-9]. These previously published applications of RCR to experimental data have involved the analysis of diseased states. Here, we apply RCR to evaluate the biological process of cell proliferation in normal, non-diseased pulmonary cells. The lung-focused Cell Proliferation Network described in this paper was constructed and evaluated by applying RCR to published gene expression profiling data sets associated with measured cell proliferation endpoints in lung and related cell types.

The Cell Proliferation Network reported here provides a detailed description of molecular processes leading to cell proliferation in the lung based on causal relationships obtained from extensive evaluation of the literature. This novel pathway model is comprehensive and integrates core cell cycle machinery with other signaling pathways which control cell proliferation in the lung, including EGF signaling, circadian clock, and Hedgehog. This pathway model is computable, and can be used for the qualitative systems-level evaluation of the complex biological processes contributing to cell proliferation pathway signaling from experimental gene expression profiling data. Construction of additional pathway models for key lung disease processes such as inflammatory signaling and response to oxidative stress is planned in order to build a comprehensive network of pathway models of lung biology relevant to lung disease. Scoring algorithms are under development to enable application of this Cell Proliferation Network and other pathway models to the quantitative evaluation of biological impact across data sets for different lung diseases, time points, or environmental perturbations.

## Results and Discussion

### Cell Proliferation Network construction overview

**Figure 1:**
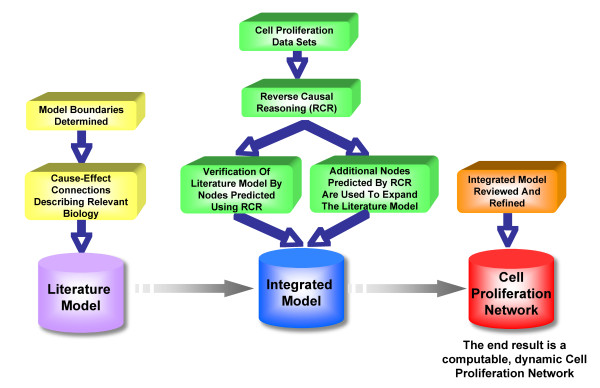
**Schematic diagram showing the iterative workflow used to create the Cell Proliferation Network**. The Cell Proliferation Network contains two components. The Literature Model (purple cylinder) was constructed from causal connections (within the tissue context and biological mechanism model boundaries) from the Selventa Knowledgebase. The content of the Literature Model was verified by performing Reverse Causal Reasoning (RCR) on four publicly available proliferation relevant data sets. In addition, the Literature Model was augmented with additional proliferation relevant RCR-derived nodes in this analysis, creating the Integrated Model. The Cell Proliferation Network (red cylinder) resulted from a comprehensive review of the Integrated Model.

The construction of the Cell Proliferation Network was an iterative process, summarized in Figure 1. The selection of biological boundaries of the model was guided by literature investigation of signaling pathways relevant to cell proliferation in the lung. Causal relationships describing cell proliferation (Additional file 1) were added to the network model from the Selventa Knowledgebase (a unified collection of over 1.5 million elements of biological knowledge captured from the public literature and other sources), with those relationships coming from lung or lung-relevant cell types prioritized (see Network boundaries, assumptions, & structure). To avoid unintentional circularity, we excluded the causal information from the specific evaluation data sets used in this study when building and evaluating the network. These data sets were analyzed using Reverse Causal Reasoning (RCR), a method for identifying predictions of the activity states of biological entities (nodes) that are statistically significant and consistent with the measurements taken for a given high-throughput data set (see Materials and Methods for additional detail). The RCR prediction of literature model nodes in directions consistent with the observations of cell proliferation in the experiments used to generate the gene expression data verified that the model is competent to capture mechanisms regulating proliferation. Additionally, proliferation-relevant nodes predicted by RCR which were not already represented in the literature model were used to extend the model. Using this approach, we generated a more comprehensive network with nodes derived from existing literature, as well as nodes derived from cell proliferation data sets, to create an integrated Cell Proliferation Network (see Network Verification and Expansion).

### Cell Proliferation Network content

**Figure 2:**
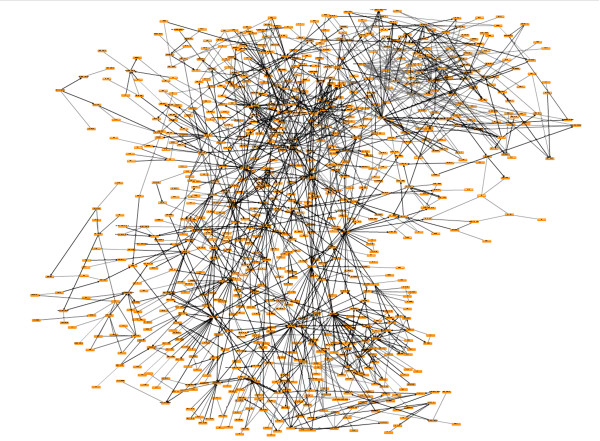
**The Cell Proliferation Network**. A graphical view of the entire Cell Proliferation Network, containing 848 nodes (orange rectangles) and 1597 edges (grey and black lines interconnecting nodes).

**Figure 3:**
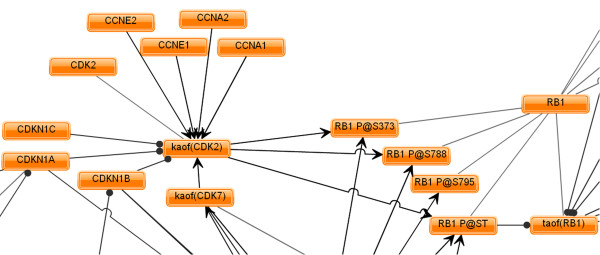
**Detail of a sub-network of the Cell Proliferation Network showing regulation and downstream effects of CDK2 kinase activity**. Nodes in the Cell Proliferation Network are represented by orange rectangles (e.g. CCNE1 or kaof(CDK2) (kinase activity of CDK2)). Edges on the model (connections between nodes) are represented as lines. Non-causal edges (e.g. the relationship between CDK2 and the kaof(CDK2)) are shown in light grey lines. Causal edges are represented by dark black lines, with edges ending in arrowheads designating positive relationships (e.g. increases or activates) and edges ending in a ball designating negative relationships (e.g. decreases or inhibits). Specific phosphorylation sites are designated with the P@X representation, where X is a specific amino acid residue or residue class. For example, the kinase activity of CDK2 phosphorylates RB1 at serine (S) residue 373. In the sub-network detail, the "kaof" prefix refers to the kinase activity of a node, while the "taof" prefix refers to the transcriptional activity of a node. Figure 4 contains a key relating the prefixes shown in the sub-network detail to their biological meaning/interpretation.

**Figure 4:**
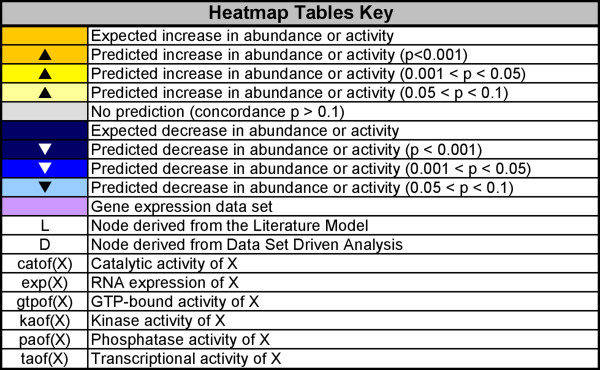
**Genstruct Technology Platform key for heatmaps**. This schedule explains the symbols and color codes used in Figures 6, 7, and 8

The Cell Proliferation Network represents a broad collection of biological mechanisms that regulate cell proliferation in the lung, and was built using a framework that is amenable to computational analyses. The Cell Proliferation Network (diagrammed in its entirety in Figure 2 and detailed in Figure 3) contains 848 nodes, 1597 edges (1091 causal edges and 506 non-causal edges (Table 1)), and was constructed using information from 429 unique PubMed-abstracted literature sources (Additional file 1). Nodes in the network are biological entities, such as the mRNA, protein, or enzymatic activity linked to a given gene; nodes may also be cellular processes such as "cell proliferation" or phases of the cell cycle. This fine-grained representation of biological entities allows for highly accurate qualitative modeling of biological mechanisms. An example can be seen from the sub-network detail in Figure 3, showing several representative network node types, including root protein nodes (CCNE1), modified protein nodes (RB1 phosphorylated at specific serine residues, represented as RB1 P@X, where X is a specific amino acid residue) and activity nodes (kinase activity of CDK2 (kaof(CDK2)) and transcriptional activity of RB1 (taof(RB1)). Figure 4 contains a key relating the prefixes (for example "kaof") shown in the sub-network detail to their biological meaning/interpretation. Edges are relationships between nodes and may be either non-causal or causal. Non-causal edges connect different forms of a biological entity, such as an mRNA or protein complex, to its base protein(s) (for example, STAT6 phosphorylated at tyrosine 641 has a non-causal relationship to its root protein node, STAT6) without an implied causal relationship. Causal edges are cause-effect relationships between biological entities, for example the increased kinase activity of CDK2 causally increases phosphorylation of RB1 at serine 373. Each causal edge is supported by a text line of evidence from a specific source reference. Additional contextual details of the relationship, such as the species and tissue/cell type in which the relationship was experimentally identified, are associated with causal edges. For this work, we used causal edges derived only from published experiments performed in human, mouse, and rat model systems, both *in vitro *and *in vivo*. This lung-focused, fully referenced Cell Proliferation Network provides the most comprehensive publicly available connectivity map of the molecular mechanisms regulating proliferative processes in the lung.

**Table 1:** Cell Proliferation Network statistics

Nodes	848
mRNAs	80

Proteins	299

Phosphoproteins	110

Activities	214

Complexes	67

Protein families	34

Biological processes	16

Proxies	15

Other	13

Total Edges	1597

Causal Edges	1091

Unique PMIDs	429

Summary of relevant statistics describing the content of the Cell Proliferation Network

### Network boundaries, assumptions, and structure

When constructing the model using content derived from the Selventa Knowledgebase, some initial boundary conditions and a priori assumptions relating to tissue context and biological content were established to constrain the substance of the model to its most salient details.

### Tissue context boundaries

Our goal was to build a network model that captures the biological mechanisms controlling cell proliferation in non-diseased mammalian lung. To maintain the focus of the network on these elements, we determined and applied a set of rules for selecting network content. Ideally, all causal relationships comprising the network would be supported by published data from experiments conducted in non-diseased human, mouse, or rat whole lung. Thus, causal relationships with literature support coming from whole lung or normal lung cell types (e.g. bronchial epithelial cells, alveolar type II cells, etc.) were prioritized. However, in many cases, the results of the relevant detailed experiments have not been published. Thus, as a second priority, relationships derived from cell types that are found in the normal lung (fibroblasts, epithelial/endothelial cells), but not explicitly from lung were used. The network was focused on relationships derived from experiments done in human systems, though relationships from mouse and rat were also included. Canonical mechanisms, such as the regulation of E2F transcription factor family members by the retinoblastoma protein RB1, were included in the network even if literature support explicitly demonstrating the presence of the mechanism in lung-related cells was not identified. It was assumed that the individual relationships within canonical mechanisms (for example CDKN1A inhibiting the kinase activity of CDK2) can occur in the lung. However, if canonical relationships with specific lung contexts were found in the literature, they were used. If needed for completing critical mechanisms within the network, relationships with other tissue contexts were used, provided they reflected proliferative processes that can occur in the normal lung. Causal relationships derived from embryonic tissue contexts were included, as the embryonic lung represents a model for non-diseased lung cell proliferation [10,11]. As a general rule, the use of causal relationships with tissue contexts from immortalized cell lines was limited to providing the molecular details for mechanisms in the network when these specific relationships were not available from normal cells; immortalized cell lines are highly amenable to experimental manipulation and are thus a valuable system for identifying signaling pathway details that are most likely conserved in normal cells. Relationships with tissue contexts derived from tumors or other diseased tissues were used sparingly in order to focus the content of the network to the pathways involved in normal lung cell proliferation.

### Biological mechanism boundaries

The Cell Proliferation Network represents the biological mechanisms leading to cell proliferation in a specific organ, the lung. Thus, biological boundaries were designed to focus the network on the cellular processes and signaling pathways with a described role in regulating lung cell proliferation, with a particular emphasis on the proximal connections to core cell cycle machinery. Following an exhaustive search of the literature, a set of pathways were selected for inclusion, while other pathways with less direct relevance for proliferation were excluded, creating the mechanistic biological boundaries of the network. These biological mechanism boundaries were used to ensure that the Cell Proliferation Network represented the most relevant proliferative mechanisms that occur in the non-diseased lung.

Cell proliferation can be directly or indirectly influenced by a wide range of factors, including external biological stimuli (e.g. growth factors) and internal metabolic alterations (e.g. ATP homeostasis). The broad range of factors that can influence cell proliferation, coupled with the observation that many proteins involved in regulating cell proliferation have varying degrees of biological promiscuity (e.g. p53 also regulates the DNA damage response and apoptosis [12,13]), necessitated some additional delineations framing the biological boundaries of the network. Therefore, in addition to defining the biological content included in the network, certain processes and pathways were explicitly excluded. Specifically, inflammatory cytokine signaling, the p53-dependent DNA damage response, and pathways regulating the induction of/escape from apoptosis were not included in the network. Finally, components of the core replication, transcription, and translation machinery (DNA/RNA polymerases, ribosomes, etc.) were considered outside the boundaries of the network.

**Figure 5:**
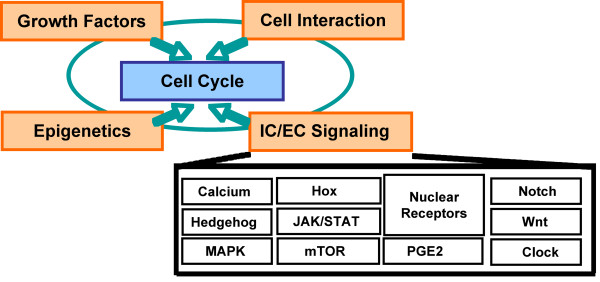
**Schematic overview of the "building block" framework used to construct the network**. Five "building blocks", each representing areas of biology known to be important for regulating lung cell proliferation, were used as a conceptual guide to construct the network. The Cell Cycle, containing the signaling elements most proximal to driving entry/exit from a proliferative state, was the central block, while connections from four other peripheral building blocks (Growth Factors, Cell Interaction, Epigenetics and Intra- and Extracellular (IC/EC) Signaling) to the Cell Cycle block were also used to construct the network. Due to the size and complexity of the IC/EC block, it was further divided into 11 sub-networks, each focused on a distinct area of cellular signaling related to regulating lung cell proliferation.

The Cell Proliferation Network was constructed in a modular fashion using a "building block" framework in which a core Cell Cycle building block is connected to additional biological pathways that contribute to cell proliferation in the lung (Figure 5). These supporting blocks are peripheral to, but connected to the core cell cycle machinery regulating proliferative processes in the lung. Briefly, the five building blocks are:

#### Cell Cycle

Includes canonical elements of the core machinery regulating entry and exit from the mammalian cell cycle, including but not limited to cyclin, CDK, and E2F family members.

#### Growth Factors

Includes common extracellular growth factors involved in regulating lung cell proliferation, namely EGF, TGF-beta, VEGF, and FGF family members. The EGF family members EGF and TGF-alpha play critical roles in regulating the proliferation of airway epithelial cells through EGF receptor activation [14,15]. FGF7 and FGF10, largely through activation of FGFR2-IIIb signaling, stimulate lung epithelial cell proliferation as well as regulate branching morphogenesis in the developing lung [16,17]. VEGF, a key regulator of normal angiogenesis and involved in regulating proliferation of human fetal airway epithelial cells, [18] was also included.

#### Intra- and Extracellular (IC/EC) Signaling

This block contains diverse elements of the common intra- and extracellular pathways involved in mediating lung cell proliferation, including the Hedgehog, Wnt, and Notch signaling pathways. Hedgehog signaling regulates cell proliferation and branching morphogenesis in the developing mammalian lung [19,20]. Similarly, Notch signaling controls lung cell proliferation as well as differentiation [21]. Elements of the Wnt signaling pathway are important for mediating the proliferative processes seen following lung injury [1]. The remaining areas covered by this building block are calcium signaling, MAPK, Hox, JAK/STAT, mTOR, prostaglandin E2 (PGE2), Clock, and nuclear receptor signaling as relevant to lung cell proliferation.

#### Cell Interaction

Includes the signal transduction pathways leading to cell proliferation that originate from the interactions of common cell adhesion molecules (including ITGB1 complexes with ITGA1-3 chains) and extracellular matrix components (specifically collagen, fibronectin, and laminin).

#### Epigenetics

Includes the main known epigenetic modulators of lung cell proliferation including the histone deacetylase (HDAC) family and DNA methyltransferase (DMT) family member DNMT1. For this block, connections from these epigenetic mediators to the core cell cycle components (e.g. CCND1, CDKN2A) were prioritized.

### Network verification and expansion

#### Selection of published cell proliferation transcriptomic data sets for verification

In order to verify the content of the network, we used publicly available data from experiments in which cell proliferation was modulated in the lung or lung relevant cell types. Specifically, we analyzed transcriptomic data sets using Reverse Causal Reasoning (RCR), which identifies upstream controllers ("hypotheses") that can explain the significant mRNA State Changes in a given transcriptomic data set. Upon completing the literature model, a search was initiated for transcriptomic data sets to verify and expand the model using public data repositories such as GEO (Gene Expression Omnibus) and ArrayExpress. The ideal data set would have been collected from either whole lung or a specific untransformed lung cell type, involves a simple perturbation affecting cell proliferation (but only minimally affecting biological processes outside of proliferation such as apoptosis), have cell proliferation phenotypic endpoint data (e.g. cell proliferation assays, or immunostaining for markers of cell proliferation), and have raw data available with at least three biological replicates for each sample group to clearly identify statistically significant changes in gene expression. Although this ideal data set was not found, these criteria were used to identify four "next best" data sets for these purposes (Table 2). The EIF4G1 data set (GSE11011) examines gene expression changes associated with decreased cell proliferation resulting from EIF4G1 knockdown in human breast epithelial cells (MCF10A cell line) [22]. The RhoA data set (GSE5913) examines gene expression changes associated with increased cell proliferation in NIH3T3 mouse fibroblasts, caused by the introduction of the dominant activating RhoA Q63L mutation [23]. The CTNNB1 data set (PMID 15186480) examines gene expression changes resulting from expression of constitutively active Ctnnb1-Lef1 fusion protein in embryonic lung, which causes increased cell proliferation and altered cell differentiation [24]. Finally, the NR3C1 data set (E-MEXP-861) examines gene expression changes resulting from glucocorticoid receptor (GR or NR3C1) knockout in embryonic mouse lung, which leads to increased cell proliferation [25]. The EIF4G1 and RhoA experiments were not performed in lung-derived cells (they were done in breast epithelial and fibroblast cell lines, respectively), however were used in the network construction process due to 1) the proximity of the perturbation used to modulate cell proliferation to the mechanisms which are known to occur in lung cells and 2) the knowledge that these cell types (epithelial cells and fibroblasts) can be found in the normal lung. By this reasoning, even though the gene expression studies in the EIF4G1 and RhoA data sets were not performed in lung cells directly, we expected to observe the shared or common mechanisms regulating proliferation in the cell types commonly found in lung tissue.

**Table 2:** Data sets analyzed for verification and expansion of the cell proliferation literature model

Data Set	EIF4G1	RhoA	CTNNB1	NR3C1
Data Set ID	GSE11011	GSE5913	PMID15186480	E-MEXP-861

PubMed ID	18426977	17213802	15186480	17901120

Perturbation	EIF4G1 siRNA	RhoA Q63L	constitutive beta-catenin-LEF-1	glucocorticoid receptor null

Control Samples	3 control	8 control	3 control	3 control

Experimental Samples	3 siRNA	7 transfected	3 transgenic	3 null

Microarray Platform	Affymetrix Human Genome U133A 2.0	Affymetrix Mouse Genome U74A v2	Affymetrix Mouse Genome 430A	GE Healthcare CodeLink Mouse Whole Genome Bioarray

Tissue	MCF10A cells	NIH3T3 cells	day 18.5 embryonic lung	day 18.5 embryonic lung

Species	human	mouse	mouse	mouse

# State changes	367	1153	645	144

### Reverse Causal Reasoning on transcriptomic data sets identifies proliferative mechanisms and verifies the literature model

**Figure 6:**
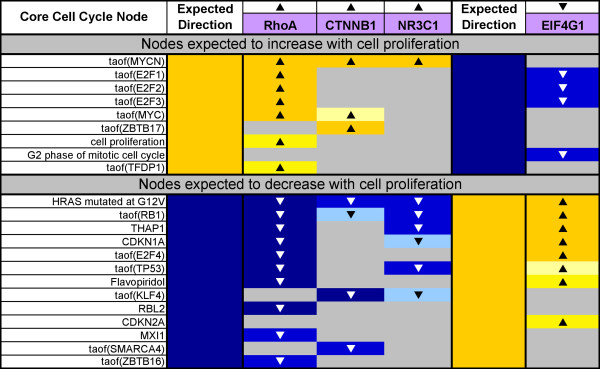
**Cell cycle block hypotheses predicted in consistent directions with observed cell proliferation**. The expected direction of a prediction in the table is based on the known biological role(s) for a given hypothesis, and is shown for the core Cell Cycle building block. The arrows above the data set names (RhoA, CTNNB1, NR3C1, and EIF4G1) denote the direction in which proliferation was observed to change in the respective experiments.

**Figure 7:**
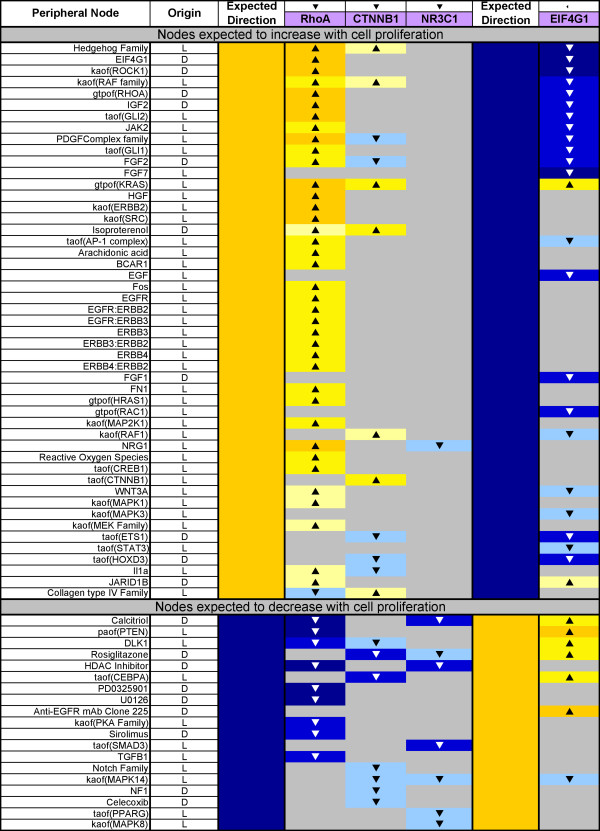
**Peripheral building block hypotheses predicted in consistent directions with observed cell proliferation**. The expected direction of a prediction in the table is based on the known biological role(s) for a given hypothesis, and is shown for the peripheral building blocks (orange and white blocks in Figure 5). The arrows above the data set names (RhoA, CTNNB1, NR3C1, and EIF4G1) denote the direction in which proliferation was observed to change in the respective experiments.

We performed RCR analysis on each of these four cell proliferation transcriptomic data sets and evaluated the resulting hypotheses. Foremost, we assessed whether nodes in the cell proliferation literature model were predicted as hypotheses in directions consistent with their biological roles (e.g. was the transcriptional activity of E2F1, a known transcriptional activator of genes required for cell cycle progression [26], predicted increased in data sets where cell proliferation was observed increased?). This analysis served as a means to verify the content of the literature model, as hypothesis predictions for a literature node can be taken as evidence that the particular proliferation-relevant mechanism(s) are operating in the context of known experimentally modulated cell proliferation. Figure 4 shows the Genstruct^® ^Technology Platform heatmap key for Figure 6, Figure 7, and 8. Figure 6 and 7 show the RCR-predicted hypotheses from the four verification data sets which were present in the literature model. Figure 6 shows the predictions for many nodes in the core Cell Cycle block, including increased E2F1, 2, and 3 activities, consistent with their published role in regulating cell proliferation in lung relevant cell types [27,28]. In addition, predictions for increased MYC activity in the RhoA and CTNNB1 data sets are consistent with the reported role of MYC in positively regulating cell proliferation in lung and lung relevant cell types [29,30]. In addition to predictions for increased activity of positive cell proliferation mediators in data sets where cell proliferation was experimentally induced to increase, RCR also predicted decreased activities of negative regulators of proliferation. Specifically, decreases in the transcriptional activity of RB1 and E2F4, both known negative regulators of cell cycle progression [31,32], were predicted in multiple data sets. Likewise, decreases in the abundance of CDKN1A or CDKN2A, cell cycle checkpoint proteins with potent anti-proliferative effects, were also predicted in all three data sets where proliferation was observed increased (Figure 6) [33,34]. One interesting prediction was that of decreased HRAS mutated at G12V. Although HRAS activity would be expected to increase, the HRAS G12V mutation leads to oncogene-induced senescence [35]; therefore, this hypothesis likely reflects a transcriptional signature of decreased senescence.

**Figure 8:**
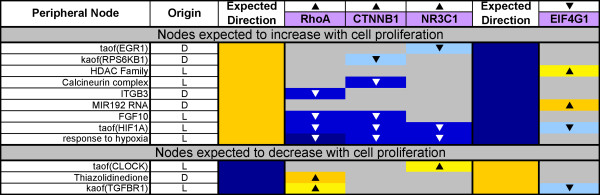
**Peripheral building block hypotheses predicted in inconsistent directions with observed cell proliferation**. The expected direction of a prediction in the table is based on the known biological role(s) for a given hypothesis, and is shown for all nodes in model. However, because there were no hypotheses in the core Cell Cycle block that were predicted in inconsistent directions, the hypotheses shown in this table are all from peripheral blocks (orange and white blocks in Figure 5). The arrows above the data set names (RhoA, CTNNB1, NR3C1, and EIF4G1) denote the direction in which proliferation was observed to change in the respective experiments.

RCR-predicted hypotheses appearing within the Cell Cycle block of literature model nodes provided verification that the proximal mechanisms regulating cell proliferation were 1) correctly present in the literature model and 2) detectable using this computational approach. However, equally important were the predictions for nodes in the peripheral building blocks, which 1) identify additional mechanistic detail for the proliferative pathways modulated and 2) can be used together with the hypothesis predictions in the core Cell Cycle block to assess the coverage of the literature model by all four data sets (see "Evaluation of the Cell Proliferation Network"). For the purposes of highlighting the peripheral mechanisms involved in lung cell proliferation, hypotheses within the growth factors building block were especially well represented, including predicted increases in PDGF, FGFs 1, 2 and 7, HGF, and EGF and its receptors (Figure 7). In particular, hypotheses for decreased FGF1 and FGF7 (also known as KGF (keratinocyte growth factor)) were predicted in the EIF4G1 data set, directionally consistent with the experimental observation of decreased proliferation observed in MCF10A epithelial cells. Both FGF1 and FGF7 are critical for promoting epithelial cell proliferation in the developing respiratory epithelium [36,37]. Several EGF receptor complexes and their ligands, which also play central roles in regulating normal lung cell proliferation, were also predicted as hypotheses in this analysis [38-40]. These hypotheses were especially noticeable in the RhoA data set, which used NIH3T3 cells as an experimental model. Although NIH3T3 cells normally express low levels of EGF family receptors and are minimally responsive to EGF, RhoA activation has been shown to decrease EGFR endocytosis, which could lead to increased levels of EGF family responsiveness in RhoA overexpressing cells [41-44]. Hypotheses from many of the other blocks of the cell proliferation literature model are also predicted in directions consistent with the observed direction of cell proliferation in the four data sets, with nodes from the cell interaction (FN1, SRC activity), MAPK signaling (MAPK 1/3 activity, MEK family), Hedgehog (Hedgehog family, GLI 1/2 activity), and WNT/beta-catenin (CTNNB1 activity, WNT3A) blocks being particularly well represented.

Despite the large number of RCR-derived hypotheses corresponding to nodes in the Cell Proliferation Network predicted in directions consistent with increased cell proliferation, some showed a different pattern. Figure 8 shows the RCR-derived hypotheses corresponding to nodes in the Cell Proliferation Network that were predicted in a direction that is opposite to what we expected based on their literature-described roles in regulating lung cell proliferation. Many of these hypotheses are pleiotropic signaling molecules, which are involved in other processes in addition to proliferation, and may result from the perturbation of non-proliferative areas of biology in the data sets examined. For example, the "response to hypoxia" and transcriptional activity of HIF1A (taof(HIF1a)) predictions may be more indicative of angiogenesis than proliferation. Additionally, some of these hypotheses may be predicted in unexpected directions due to feedback mechanisms or other forms of regulation. Finally, these predictions may also result from alternative activities of these signaling molecules that have not been described in the literature, such as the microRNA MIR192, which is still in the early stages of research into its functions. It is important to note that none of the hypotheses predicted in unexpected directions are nodes in the core Cell Cycle block, an observation that further verifies the cell proliferation literature model.

This analysis supported the model as an accurate and comprehensive representation of cell proliferation in the lung. Predictions for nodes in the core Cell Cycle and Growth Factor blocks are especially robust, consistent with the key role these elements play in cell proliferation. The analysis also confirms the ability of RCR to predict proliferative mechanisms based on transcriptomic data from multiple, independent data sets. Therefore, the proliferation literature model (and the framework used to create it) appears to be very well-suited for the evaluation of mechanisms guiding lung cell proliferation using gene expression microarray data sets.

#### Expansion of the literature model using data set-derived nodes to create the integrated model

In addition to verifying the cell proliferation literature model, RCR on the four cell proliferation data sets was used to identify other mechanisms impacting cell proliferation in the lung. The prediction of a hypothesis in a cell proliferation data set may suggest involvement in proliferation; however, they may also reflect other biological processes that are affected by the experimental perturbations in these data sets. Therefore, each of the hypotheses predicted by RCR in these four data sets that were not already included in the model was investigated to determine its role in lung proliferation. Hypotheses that were determined to play a role in lung proliferation based on surveys of the literature were then further examined to determine how they could best be integrated into the existing literature model. These nodes (33 in total) were then added to the model, creating a more robust and comprehensive network of lung proliferation. The literature model supplemented with these data set-derived nodes is referred to in this paper as the integrated Cell Proliferation Network, as it takes into account not only known proliferative mechanisms operating in the lung from the literature, but also additional mechanisms determined to play a role in lung cell proliferation identified by RCR on cell proliferation data sets. For example, the transcriptional activity of Zbtb17 (MIZ-1), was predicted to be increased in the CTNNB1 data set (Figure 6). MIZ-1 is ubiquitously expressed during embryonic development and has the ability to induce growth arrest [45]. Recently, it has been reported that the physical interaction of MIZ-1 with MYC blocks the ability of MIZ-1 to induce growth arrest, partially through removing the ability of MIZ-1 to activate p15INK4b gene expression [46]. While Zbtb17 is known to influence the transcriptional activity of MYC [47], and cell proliferation in other cell types, it does not yet have a direct literature-described role in regulating normal lung cell proliferation. The data set-derived nodes added to the literature model as a result of their prediction as hypotheses in the cell proliferation data sets are designated in Figure 6 and 7 by the "D" in the 'Origin' column.

The content of the Knowledgebase (the substrate used to build the proliferation network) used in this study is constantly updated with the latest scientific information. As such, the proliferation model itself is dynamic, and has the flexibility to represent a contemporary view of lung cell proliferation as scientific knowledge advances. RCR prediction of a given node using gene expression data sets requires a minimum of four observed RNA expression changes that are consistent with the predicted change in node activity in the Knowledgebase. Thus, one reason that a network node may not be predicted as a hypothesis using RCR on the cell proliferation data sets is that the Knowledgebase contains too few causal connections from the node to downstream RNA expressions. To address this, we took advantage of the dynamic property of the Knowledgebase to perform targeted knowledge curation around specific nodes in order to increase the likelihood of detecting them as hypotheses using RCR. The extent of these curation efforts was limited to a subset of nodes in the proliferation network, however the structural framework used in the construction of this network allows for additional knowledge to be incorporated in the future.

### Evaluation of the Cell Proliferation Network

In order to evaluate the content of the Cell Proliferation Network we assessed the coverage of network nodes predicted by RCR (on the four cell proliferation data sets) as a percentage of total network nodes that were capable of being predicted. In all, 229 of the 848 nodes in the Cell Proliferation Network met the minimum criteria to be predicted changed by RCR (i.e. there were four or more observed RNA expression changes consistent with the predicted change contained in the Knowledgebase) and are termed the "possible nodes". Of these 229 "possible nodes", RCR predicted changes in 102 (45%) in at least one of the four cell proliferation data sets. Seventy one (31%) were predicted based on the RhoA data set alone, while 31 (14%), 19 (8%) and 47 (21%) were predicted based on the CTNNB1, NR3C1, and EIF4G1 data sets, respectively. Notably, many of the nodes for which a prediction was not possible exert their influences on proliferation via non-transcriptional events, such as phosphorylation, degradation, etc., or have limited published information regarding their influences on gene expression. As such, these nodes would be more likely predicted to increase or decrease when using a combination of systems biology data types (e.g. gene expression and phosphoproteomic arrays). These results further verify the Cell Proliferation Network, as well as the method of using RCR to predict proliferative mechanisms using systems biology data.

As noted in the "Network verification and expansion" section, the ideal publicly available data set for verifying the network would have adhered to collection of quality control criteria including 1) non-diseased lung tissue focus, 2) simple perturbation of primarily cell proliferation (as opposed to other biological processes such as apoptosis), 3) relevant endpoint data, and 4) statistical soundness. The data sets used for evaluating the model were chosen because they all met criteria 2-4 detailed above, and were also done in lung cell relevant contexts. In fact, two of the data sets were derived from experiments done in embryonic lung, and two were done in cell types that best approximated the biology occurring in lung cells.

The network described here is the first step in the larger objective of creating an integrated network of lung biology. The Cell Proliferation Network portrays the signaling pathways involved in normal lung cell proliferation with expanded coverage relative to existing representations. However, it relates only a subset of the processes involved in many complex lung diseases. For example, lung cancer is a disease of uncontrolled cell proliferation, but also involves response to DNA damage and apoptosis evasion components, among others [48,49]. Similarly, chronic lung diseases such as asthma and COPD involve not only alterations in the proliferative aspects of cell populations, but also profound alterations in the inflammatory response [50,51]. In this light, a truly systemic evaluation of diseases such as these will require networks that cover multiple biological processes in a lung focused and interconnected manner. As such, the Cell Proliferation Network is the first of several planned networks that will be built over the coming months to capture the known universe of biological processes relevant for lung disease in a comprehensive, centralized, and computable structure (Additional file 2 corresponds to the entire Cell Proliferation Network in a computable format).

## Conclusions

Cell proliferation is a complex biological process with relevance to several common lung diseases. Modern systems biology data, such as transcriptomics, are useful in unraveling the detail embedded in complex processes like cell proliferation, but require the appropriate tools. The publicly available lung focused Cell Proliferation Network described here represents the most comprehensive and fully referenced mechanistic representation of the signaling pathways that regulate normal lung cell proliferation in existence, and is compatible with analysis using systems biology data. The adaptable and computable structure of the network makes it a useful tool for a wide variety of research investigators across broad scientific disciplines.

## Methods

### Knowledgebase and Knowledge Assembly Models

**Figure 9:**
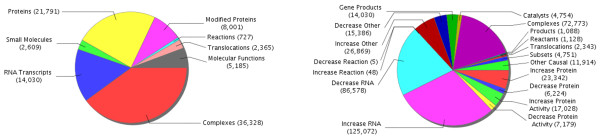
**Pie charts showing the nodes (biological entities, left) and causal edges (causal relationships, right) contained in the Human Knowledge Assembly Model (KAM)**. Knowledge Assembly Models are collections of nodes (biological entities) and causal edges (causal relationships) and are used as substrates for Reverse Causal Reasoning (RCR). The number of nodes and causal edges contained in the Human KAM for each sub-category is shown in parentheses. These pie charts are a snapshot of the Human KAM as of August 18, 2010; the KAM is continually being expanded and refined. KAMs are also maintained for Mouse and Rat, and additional custom KAMs can also be created. Every KAM represents a subset of the global Selventa Knowledgebase.

The nodes and edges comprising the Cell Proliferation Network were added to the model from the Selventa Knowledgebase, a comprehensive repository containing over 1.5 million nodes (biological concepts and entities) and over 7.5 million edges (connections between nodes). The Selventa Knowledgebase is derived from peer-reviewed scientific literature as well as other public and proprietary databases. In addition to containing a vast collective of causal relationships derived from healthy tissues, the Knowledgebase is particularly enriched in disease areas such as inflammation, metabolic diseases, cardiovascular injury, liver injury and cancer. Knowledge Assembly Models (KAMs) are subsets of the global Selventa Knowledgebase designed to facilitate reasoning and computation (Figure 9).

The human KAM is the set of causal assertions from human sources that has been augmented with orthologous causal assertions derived from either mouse or rat sources, and is competent for RCR (see Reverse Causal Reasoning (RCR): Automated Hypothesis Generation). Similarly, the mouse KAM is the set of causal assertions derived from mouse sources that has been augmented with orthologous causal assertions derived from either human or rat sources. Each KAM contains approximately 90,000 total nodes and 400,000 total edges, incorporating information from over 35,000 distinct citations. An example causal assertion is increased transcriptional activity of EGR1 (early growth response 1) causing an increase in the expression of CCND1 (cyclin D1). Each such causal assertion has a specific scientific citation, and the assembled collection of these causal assertions is referred to as either the human or mouse KAM in this paper. The Selventa Knowledgebase and KAMs provide a framework for developing computable, qualitative models of specific areas of biology.

When analyzing public gene expression data sets for the construction and verification of the network, the full human KAM was used as the substrate for RCR; however the Cell Proliferation Network itself (the nodes and edges that make up the physical network connectivity) reflects a subset of all the causal assertions in the human KAM.

### Reverse Causal Reasoning (RCR): Automated hypothesis generation

Reverse causal reasoning (RCR) was used to verify and expand the Cell Proliferation Network using cell proliferation experiments with publicly available transcriptomic profiling data. RCR interrogates a species-specific KAM to identify upstream controllers of the RNA State Changes (see Analysis of transcriptomic data sets section) observed in the data set. These upstream controllers are called "hypotheses", as they are statistically significant potential explanations for the observed RNA State Changes. Hypothesis generation is performed automatically by a computer program that utilizes the KAM to identify hypotheses that explain the input RNA State Changes, prioritized by multiple statistical criteria. The substrate for analysis of RNA State Changes observed in the cell proliferation data sets is a species-specific KAM, which is derived from the global Selventa Knowledgebase. For the EIF4G1 data set, the human KAM was used, while the mouse KAM was used for the RhoA, CTNNB1, and NR3C1 data sets.

Each hypothesis is scored according to two probabilistic scoring metrics, richness and concordance, which examine distinct aspects of the probability of a hypothetical cause explaining a given number of RNA State Changes (see next section). Richness is the probability that the number of observed RNA State Changes connected to a given hypothesis could have occurred by chance alone. Concordance is the probability that the number of observed RNA State Changes that match the directionality of the hypothesis (e.g., increased or decreased kinase activity for a kinase, increased or decreased transcriptional activity for a transcription factor, etc.) could have occurred by chance alone. A scored hypothesis is considered to be statistically (although not necessarily biologically) significant if it meets richness and concordance p-value cutoffs of 0.1. Following automated hypothesis generation (which can generate hundreds of hypotheses for a given data set analysis), each scored hypothesis meeting the minimum statistical cutoffs for richness and concordance is evaluated and prioritized by a group of scientists based on its biological plausibility and relevance to the experimental perturbation used to generate the data. Evaluation and prioritization was based on multiple criteria, including the mechanistic proximity of the hypothesis to non-diseased lung biology and evidence that the hypothesis is present or has activity in normal lung or lung-related cells. When constructing this network, each hypothesis was collaboratively evaluated by teams of scientists from both Philip Morris International (PMI) and Selventa. For a more comprehensive and detailed explanation on hypothesis scoring and evaluation, please refer to [52].

Many hypotheses identified using RCR on the cell proliferation data sets were already represented in the literature model; those that were not represented in the literature model were investigated by evaluation of their biological relevance to the lung context and whether they are causally linked to phenotypes and processes relevant to cell proliferation in the literature. Hypotheses meeting the above criteria were then added to the literature model as data set-driven nodes, creating the integrated network model. Thus, RCR allowed for verification, testing, and expansion of the Cell Proliferation Network using publicly available proliferation data sets.

### Analysis of transcriptomic data sets

Four previously published cell proliferation data sets, GSE11011 (EIF4G1), GSE5913 (RhoA), PMID15186480 (CTNNB1), and E-MEXP-861 (NR3C1), were used for the verification and expansion of the Cell Proliferation Network (Table 2). These data sets was chosen for a variety of reasons, including 1) the relevance of the experimental perturbation to modulating the types of cell proliferation that can occur in cells of the normal lung, 2) the availability of raw gene expression data, 3) the statistical soundness of the underlying experimental design, and 4) the availability of appropriate cell proliferation endpoint data associated with each transcriptomic data set. In addition, the perturbations used to modulate cell proliferation in these experiments covered mechanistically distinct areas of the Cell Proliferation Network, ensuring that robust coverage of distinct mechanistic pathways controlling lung cell proliferation were reflected in the network. Data for GSE11011 and GSE5913 were downloaded from Gene Expression Omnibus (GEO) http://www.ncbi.nlm.nih.gov/gds, while data for E-MEXP-861 was downloaded from ArrayExpress http://www.ebi.ac.uk/microarray-as/ae/. The data from PMID15186480 was obtained from a link within the online version of the paper http://jbiol.com/content/3/3/11. Raw RNA expression data for each data set were analyzed using the "affy" and "limma" packages of the Bioconductor suite of microarray analysis tools available for the R statistical environment [53-56]. Robust Microarray Analysis (RMA) background correction and quantile normalization were used to generate microarray expression values for the Affymetrix platform data sets, EIF4G1, RhoA, and CTNNB1. Quantile normalization was applied to analysis of the GE Codelink platform data set, NR3C1. An overall linear model was fit to the data for all sample groups, and specific contrasts of interest were evaluated to generate raw *p*-values for each probe set on the expression array [57]. The Benjamini-Hochberg False Discovery Rate (FDR) method was then used to correct for multiple testing effects.

Probe sets were considered to have changed qualitatively in a specific comparison if an adjusted *p*-value of 0.05 was obtained and they had an absolute fold change greater than 1.3. An additional expression abundance filter was applied to three of the data sets; probe set differences were considered significant only if the average expression intensity was above 250 in either the control or treated group for the EIF4G1 and RhoA data sets, and above 10 for the NR3C1 data set. No abundance threshold was applied to the CTNNB1 data set. These criteria were applied to optimize State Change numbers for RCR. NetAffx version na30 feature annotation files, available from Affymetrix http://www.Affymetrix.com, were used for mapping of probe sets to genes. Genes represented by multiple probe sets were considered to have changed if at least one probe set was observed to change. Gene expression changes that met these criteria are called "State Changes" and have the directional qualities of "increased" or "decreased", i.e., they were upregulated or downregulated, respectively in response to the experimental perturbation. The number of State Changes for each data set is listed in Table 2.

## List of abbreviations used

AKT: v-akt murine thymoma viral oncogene homolog; ATP: adenosine-5'-triphosphate; BEL: Biological Expression Language; CCND1: cyclin D1; CDK: cyclin-dependent kinase; CDKN: cyclin-dependent kinase inhibitor; COPD: chronic obstructive pulmonary disease; CTNNB1: catenin, beta 1; DMT: DNA methyltransferase; DNA: deoxyribonucleic acid; E2F: E2 transcription factor; EGF: epidermal growth factor; EGR1: early growth response 1; EIF4G1: eukaryotic translation initiation factor 4 gamma, 1; FDR: false discovery rate; FGF: fibroblast growth factor; FGFR2: fibroblast growth factor receptor 2; FN1: fibronectin 1; GEO: Gene Expression Omnibus; GLI: glioma-associated oncogene family zinc finger; HDAC: histone deacetylase; HOX: homeobox family; HGF: hepatocyte growth factor; HIF1A: hypoxia inducible factor 1, alpha subunit; HRAS: v-Ha-ras Harvey rat sarcoma viral oncogene homolog; IC/EC: intracellular and extracellular; IGF2: insulin-like growth factor 2; IL1B: interleukin 1, beta; ITG: integrin; JAK: Janus kinase; KAM: Knowledge Assembly Model; KEGG: Kyoto Encyclopedia of Genes and Genomes; LEF1: lymphoid enhancer-binding factor 1; MAPK: mitogen-activated protein kinase; MCF10A: Michigan Cancer Foundation cell line 10A; MEK: mitogen-activated protein kinase kinase; MIR: micro RNA; mRNA: messenger ribonucleic acid; mTOR: mammalian target of rapamycin; MYC: v-myc myelocytomatosis viral oncogene homolog; MYCN: v-myc myelocytomatosis viral related oncogene, neuroblastoma derived; NIH3T3: National Institutes of Health cell line 3T3; NR3C1: nuclear receptor subfamily 3, group C, member 1 (glucocorticoid receptor); p53: tumor protein p53; PDGF: platelet derived growth factor; PGE2: prostaglandin E2; PMID: PubMed identifier; RB1: retinoblastoma 1; RCR: Reverse Causal Reasoning; RhoA: ras homolog gene family, member A; RMA: Robust Microarray Analysis; RNA: ribonucleic acid; SBML: Systems Biology Markup Language; SRC: v-src sarcoma (Schmidt-Ruppin A-2) viral oncogene homolog; STAT: signal transducer and activator of transcription; TGF: transforming growth factor; VEGF: vascular endothelial growth factor; WNT: wingless-type MMTV integration site family; ZBTB17: zinc finger and BTB domain containing 17 (MIZ-1).

## Competing interests

The authors declare that they have no competing interests.

## Authors' contributions

JW contributed to the network design, biological content, interpretation of results, and manuscript revision. WS contributed to the network design, biological content, interpretation of results, manuscript revision, and project co-ordination. BF, SG, NC, WH, AH, CM, RL, CP, MT, EV contributed to the network design, biological content, interpretation of results, and manuscript preparation. SE contributed to the network design, biological content, and interpretation of results. AVH, BW contributed to the biological content and interpretation of results. MM contributed to project co-ordination and manuscript preparation. AM, MP, JH contributed to system concept and supervised the project. RK contributed to system concept, network design, interpretation of results, manuscript preparation and supervised the project. All authors read and approved the final manuscript.

## Supplementary Material

Additional file 1 **The Cell Proliferation Network Model nodes-edges-evidence**. this excel file contains the complete list of evidences associated to all nodes and edges present in the Cell Proliferation Network. Click here for file

Additional file 2 **The Cell Proliferation Network Model**. this file contains the Cell Proliferation Network Model in "OWL Web Ontology Language" format. Click here for file
